# Improved Survival of Young Patients With Breast Cancer 40 Years and Younger at Diagnosis

**DOI:** 10.1200/GO.22.00354

**Published:** 2023-05-25

**Authors:** Nagi S. El Saghir, Lana E. Khalil, Joud El Dick, Rula W. Atwani, Nadine Safi, Maya Charafeddine, Ahmad Al-Masri, Bassem N. El Saghir, Maha Chaccour, Arafat Tfayli, Hazem Assi, Jaber Abbas, Zeina Ayoub, Eman Sbaity, Hiba A. Moukadem

**Affiliations:** ^1^ American University of Beirut Medical Center, Beirut, Lebanon

## Abstract

**PURPOSE:**

Around 50% of patients with breast cancer in low- or middle-income countries are younger than 50 years, a poor prognostic variable. We report the outcome of patients with breast cancer 40 years and younger.

**METHODS:**

We reviewed 386 patients with breast cancer 40 years and younger and retrieved demographic, clinicopathologic, treatment-related, disease progression, and survival data from electronic medical records.

**RESULTS:**

The median age at diagnosis was 36 years, and infiltrating ductal carcinoma was present in 94.3% of patients, infiltrating lobular carcinoma in 1.3%, and ductal carcinoma in situ in 4.4%. Grade 1 disease was present in 8.5% of patients, grade 2 in 35.5%, and grade 3 in 53.4%; 25.1% had human epidermal growth factor receptor 2 (HER2)–positive, 74.6% had hormone receptor (HR)+, and 16.6% had triple-negative breast cancer. Early breast cancer (EBC) constituted 63.6% (stage I, 22.4%; stage II, 41.2%) of patients, whereas 23.2% had stage III, and 13.2% had metastatic disease at diagnosis. Of patients with EBC, 51% had partial mastectomy and 49.0% had total mastectomy. And 77.1% had chemotherapy with or without anti-HER2 therapy. All HR+ patients received adjuvant hormonal therapy. The disease-free survival at 5 years was 72.5% and 55.9% at 10 years. The overall survival (OS) was 89.4% at 5 years and 76% at 10 years. Patients with stages I/II had an OS of 96.0% at 5 years and 87.1% at 10 years. Patients with stage III had an OS of 88.3% at 5 years and 68.7% at 10 years. The OS of patients with stage IV was 64.5% at 5 years and 48.4% at 10 years.

**CONCLUSION:**

We report survival rates of 89% at 5 years and 76% at 10 years with modern multidisciplinary management. Best results were seen in EBC: OS rates of 96% and 87% at 5 years and 10 years.

## INTRODUCTION

Breast cancer is the most common malignancy in women worldwide and is the number one cause of mortality in women.^1^ The incidence rate in the United States among White females younger than 40 years was 129 of 100,000, and they constitute <5% of the total number of breast cancers in the United States.^2^ Early-onset breast cancer before age 40 years is generally associated with a worse outcome and more advanced presentations.^2^ The tumor biology is, in general, more aggressive, and clinical outcomes are worse in the subgroup of women below age 40 years.^3^ In the United States and Europe, the median age of women with breast cancer is 63 years at onset, 19% are diagnosed below age 50 years, and only 5% are diagnosed below age 40 years,^2^ whereas in Lebanon, the Arab Countries, and most low- or middle-income countries (LMICs), 50% of patients with breast cancer are diagnosed below age 50 years,^4^ and 20.8% are below age 40 years.^5^ A study of 1,320 patients with breast cancer at the American University of Beirut Medical Center (AUBMC) found that younger (younger than 35 years) patients had a worse outcome in multivariate analysis.^6^ Furthermore, early-onset breast cancer was shown to be an independent risk factor for relapse in a retrospective study of 2,040 patients with consecutive primary invasive breast cancer as younger age at diagnosis remained a significant predictor of recurrence in multivariate analysis (*P* = .010).^3^

**CONTEXT**

**Key Objective**
Has the survival of patients diagnosed below age 40 years with breast cancer improved in clinical practice?
**Knowledge Generated**
Application of modern multidisciplinary management produced improved overall survival rates of 76% at 10 years in young patients with breast cancer. Ten-year survival rates of young patients with early breast cancer reached 87%, and patients with locally advanced and metastatic breast cancer reached rates of 68.7% and 48.4%, respectively. Two thirds of breast cancer cases are diagnosed at early stages because of early detection campaigns.
**Relevance**
Global availability and access to newer modalities of therapy and medication are essential to improve survival.

The relatively worse prognosis of early-onset breast cancer is due to a multitude of factors.^7^ Some studies suggest that breast cancer in young women is associated with more estrogen receptor/progesterone receptor (ER/PR) negativity and human epidermal growth factor receptor 2 (HER2) overexpression,^3,8^ which could partially explain the worse prognosis since such a pattern of receptor expression confers a worse outcome and survival when compared with luminal A and B when compared with their older counterparts.^3^ Furthermore, many studies have shown that breast cancer in young women tends to present with more advanced stages than in older patients. The risk of local recurrence of breast cancer was shown to be higher in younger patients in many previous studies.^2^ In addition, survival of patients with breast cancer varies globally according to resources and access to modern therapy.^9-11^ We report results from the AUBMC, a primary and tertiary care center in Lebanon.

## METHODS

### Patients and Data Collection

We reviewed the list with all the patient's hospital identification number for a sample of patients with breast cancer seen at AUBMC between 2010 and 2018. Oral consent was obtained from all patients included in the study. Using the simple random sampling method,^12^ we reviewed available complete survival data of 386 patients to be included in this analysis. After the approval of the institutional review board of AUBMC, we collected information from the medical records of the patients from the hospital's outpatient clinics and inpatient and radiation oncology departments.

### Study Variables

Variables were collected using an electronic data collection sheet, which included the following: demographic data, medical history, date of diagnosis, staging, surgical interventions, pathologic characteristics, genetic testing, treatment details, follow-up dates, recurrence details, and morbidity. Demographics included age and sex. Biopsy details included date and type of diagnosis. Pathologic data included information on tumor size, grade, histology, receptor status, and axillary lymph nodes. Hormonal receptors were determined by immunohistochemistry and were considered positive when ER, PR, or both were positive. HER2 was considered positive if +3, equivocal if +2, and negative if +1 or zero. Equivocal results were furthered checked using fluorescence in situ hybridization testing, all according to the ASCO/College of American Pathologists (CAP) guidelines.^13^ TNM staging was used according to American Joint Committee on Cancer eighth edition.^14^

Treatment data included type and duration of neoadjuvant therapy, date of surgery, breast-conserving surgery (BCS) or mastectomy, number of lymph nodes removed, number of positive lymph nodes, date of axillary lymph node dissection, adjuvant chemotherapy, radiation therapy, and hormonal therapy. Follow-up data included information about local and distant sites of recurrence. Dates and causes of death of deceased patients were recorded.

### Statistical Methods

The data were analyzed using statistical package for the social sciences (SPSS) version 25. Frequencies of all the variables in question were calculated according to univariate analysis, including frequencies, and bivariate analysis involving the calculation of two-sided *P* value using Pearson's chi square test to identify a significant correlation between the variables: median age at diagnosis, stage, grade, type, tumor size, ER and PR status, HER2 receptor status, lymph node (LN) status, surgery date and type, radiation therapy, neoadjuvant and adjuvant chemotherapy, and hormonal therapy. Disease-free survival (DFS) was defined as the time from the date of diagnosis to the date of disease recurrence or last follow-up date for censored patients. OS was defined as the time from the date of diagnosis to the date of last follow-up or date of death.

## RESULTS

A total of 386 patients below age 40 years were included (Table 1). Age ranged between 19 and 40 years. The median age was 36 years.

**TABLE 1:**
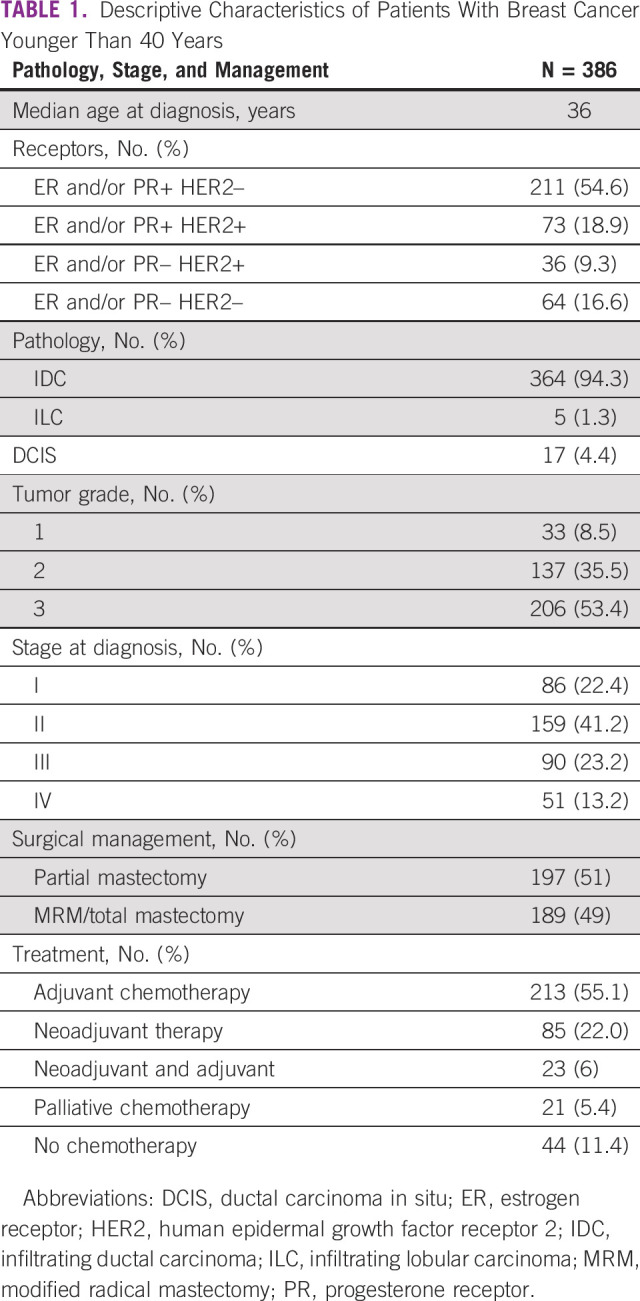
Descriptive Characteristics of Patients With Breast Cancer Younger Than 40 Years

Pathology: The majority of patients had infiltrating ductal carcinoma (IDC) constituting 94.3% of our cohort, whereas ductal carcinoma in situ (DCIS) was 4.4% and infiltrating lobular carcinoma (ILC) was 1.3%. High grade was dominant with 53.4% (206 of 386) of our patients having grade 3, 35.5% (137 of 386) having grade 2, and only 8.5% (33 of 386) having grade 1.

Hormonal receptors were positive in 288 patients (74.6% of patients), whereas HER2 was positive in 101 of 355 patients of confirmed HER2 (26.2%). Triple-negative breast cancer (TNBC) was found in 16.6% (64). HER2 testing results were missing in 31 patients.

### Stages

Of the total 386 patients, 86 patients (22.4%) were stage I, 159 patients (41.2%) were stage II, 90 patients (23.2%) were stage III, and 51 (13.2%) were stage IV at presentation. When we include recurrences, metastatic breast cancer was present in 84 patients (21.9%).

### Management

Neoadjuvant therapy was given to 85 patients (22.0%), adjuvant chemotherapy was given to 213 patients (55.1%), 23 patients (6.0%) had both neoadjuvant and adjuvant treatment, and 21 patients (5.4%) had palliative treatment. Adjuvant radiotherapy was given to 304 patients (78.8%). Adjuvant hormonal therapy was given to 274 patients (71% of the total 386). Although all 288 patients with hormone receptor–positive disease were given hormonal therapy, 14 patients did not complete at least 5 years of adjuvant hormonal therapy.

As for surgery, 51% of patients had a total mastectomy and 49% had BCS, which consisted of a partial mastectomy with radiation therapy.There were 62.5% of patients with stage I and 58.1% of patients with stage II that had BCS. Of 73 patients with stage III, 24.7% had BCS. The majority of patients had adjuvant chemotherapy, and 61.3% of patients who had neoadjuvant treatment underwent mastectomy (52 of 85).

Of patients with stage I breast cancer, 66.2% (57 of 86) had adjuvant chemotherapy, 60.3% of patients with stage II (96 of 159) had adjuvant chemotherapy, and 40.5% of patients with stage III disease (36 of 90) had adjuvant chemotherapy. Of 51 patients with stage IV breast cancer, 8 (15.8%) patients had systemic chemotherapy. Adjuvant hormonal therapy was given to all patients with positive hormonal receptors.

**FIG 1:**
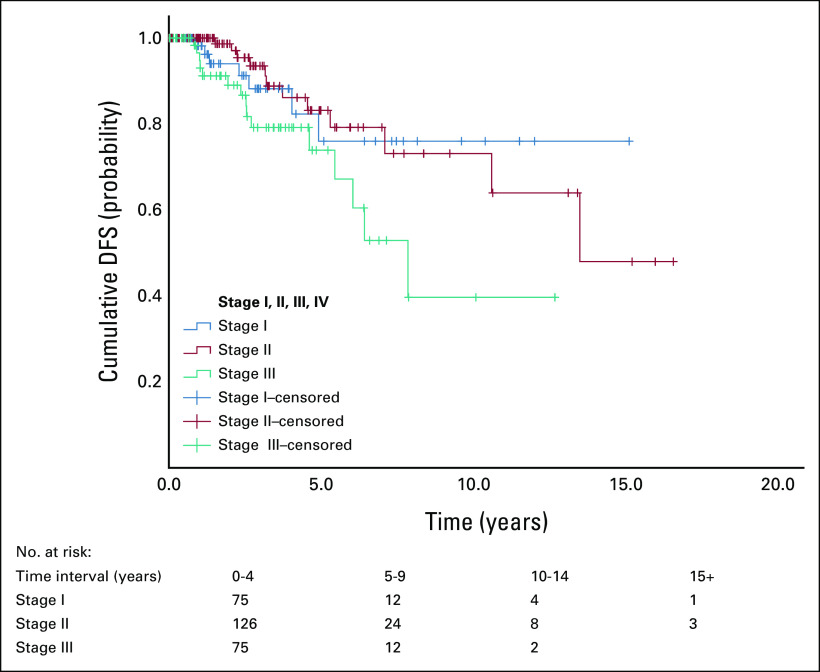
Survival by stage, DFS (n = 292). The DFS for patients with stage I breast cancer was 82.3% and 76% at 5 and 10 years, respectively, compared with 73.9% and 40% for patients with stage III at diagnosis. DFS, disease-free survival.

**FIG 2:**
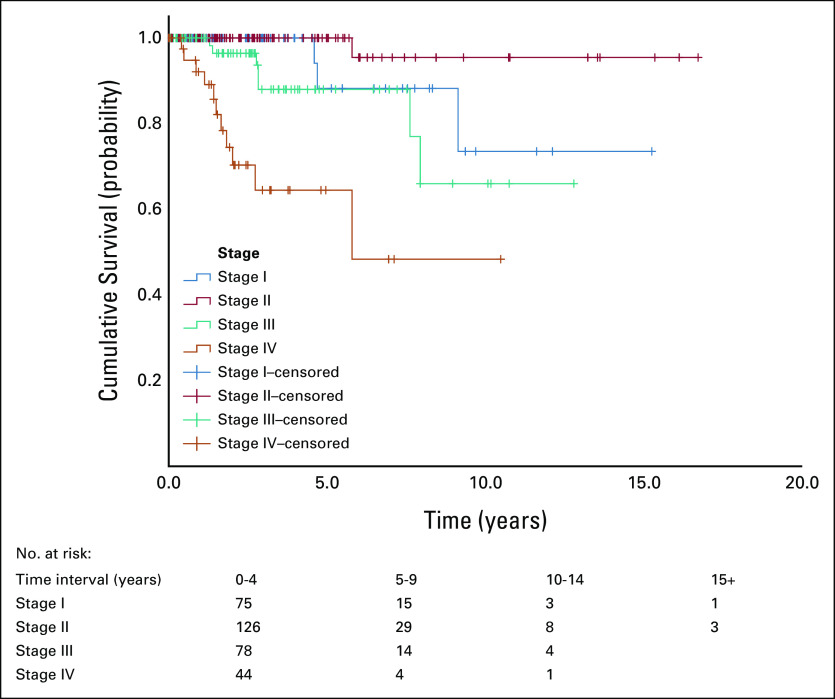
Survival by stage, OS (n = 323). The OS was 100% at 5 years and 95.5% at 10 years for patients with stage II disease at diagnosis compared with 65.5% at 5 years and 48.4% at 10 years for patients with stage IV at diagnosis. OS, overall survival.

The survival rates in Table 2 were also reflected in the survival function curves (Figs 1 and 2). Figure 1 shows that DFS was best in patients who were stage I at diagnosis and worst in those who were stage III. Overall survival (OS), however, was best in patients who were stage II at diagnosis and worst in those who were stage IV as shown in Figure 2. Survival by subtype is as follows: for luminal A patients, the 5-year survival was 90.7% and the 10-year survival was 82.6%. For luminal B patients, the 5-year survival was 87.8% and the 10-year survival was 74.2%; for HER2 patients, the 5-year survival was 82.6% and the 10-year survival was 63.0% (Table 3); for TNBC patients, the 5-year survival was 87.8% and the 10-year survival was 80.4%.

**TABLE 2:**
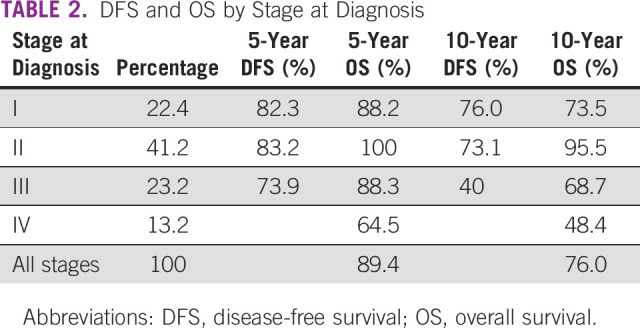
DFS and OS by Stage at Diagnosis

**TABLE 3:**
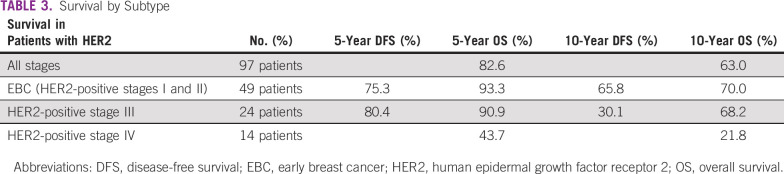
Survival by Subtype

**FIG 3:**
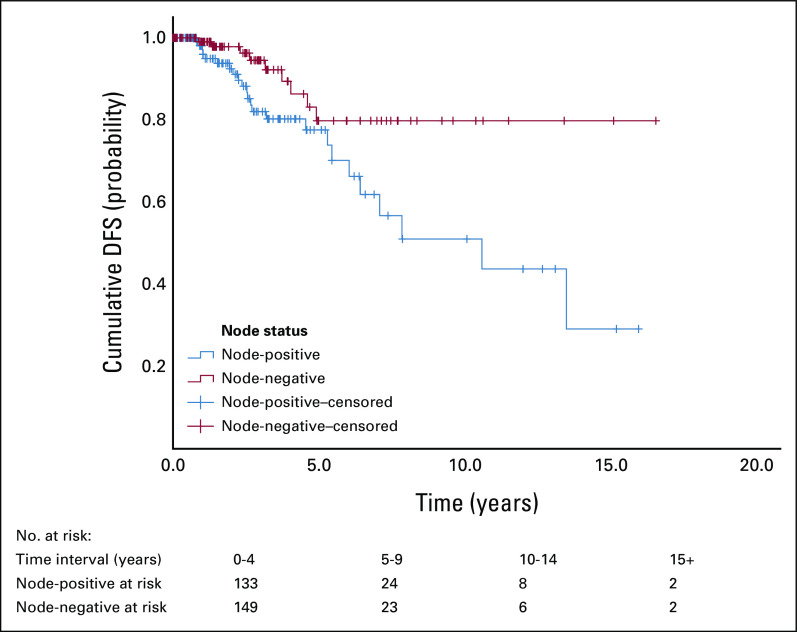
DFS by node status (n = 270). The DFS was better at 5 and 10 years in node-negative disease (83.1% and 79.8%, respectively) compared with node-positive disease (77.6% and 51%, respectively). DFS, disease-free survival.

**FIG 4:**
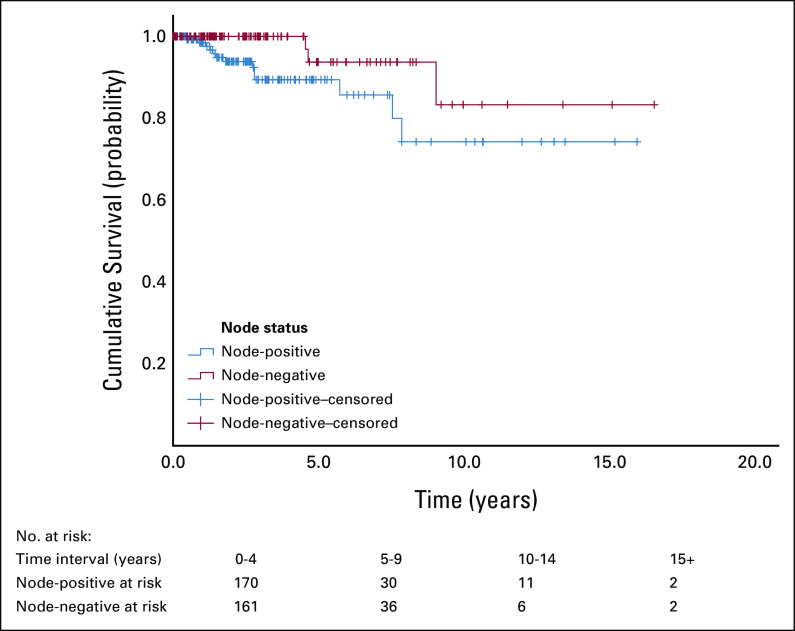
OS by node status (n = 297). The OS was better at 5 and 10 years in node-negative disease (93.8% and 83.3%, respectively) compared with node-positive disease (89.4% and 74.3%, respectively). OS, overall survival.

OS was better at 5 and 10 years in node-negative disease (93.8% and 83.3%, respectively) compared with node-positive disease (89.4% and 74.3%, respectively; Fig 4). Similarly, DFS (Fig 3) was better at 5 and 10 years in node-negative disease (83.1% and 79.8%, respectively) compared with node-positive disease (77.6% and 51%, respectively; Table 4).

**TABLE 4:**
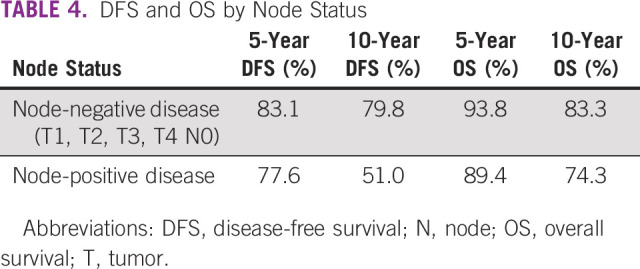
DFS and OS by Node Status

The rates in Table 4 are also reflected in Figures 3 and 4, which showed a better OS and DFS in node-negative disease when compared with node-positive disease.

## DISCUSSION

In this study, we looked at the outcome of 386 patients below age 40 years who underwent modern chemotherapy, targeted therapy, neoadjuvant/adjuvant therapy, surgery, radiation therapy, and hormonal therapy. Management was performed in Lebanon, which is considered a LMIC. We show that the majority (88.9%) had a grade 2 or 3 tumor (53.4% of patients had a grade 3 breast cancer, and 35.5% grade 2), 74% had positive hormone receptors, 26% had HER2-overexpressive tumors, and 16.6% had TNBC. Of the patients in the study, 22.4% had stage I, 41.2% had stage II, 23.2% had stage III, and 13.2% had stage IV disease at presentation. As for the pathology, the majority of our patients had IDC constituting 94%; DCIS was present in only 4.4%, and ILC in 1.3%.

The patient and tumor characteristics indicate that our cohort of patients has the features that may explain the worse outcome observed in younger patients with breast cancer. However, we report high OS rates of 89.4% at 5 years and 76% at 10 years and OS rates of 93.8% at 5 years and 83.3% at 10 years in patients with node-negative disease and 89.4% at 5 years and 74.3% at 10 years for patients with node-positive disease. These results are very encouraging and translate the advances attributed to the modern therapy with neoadjuvant, adjuvant, surgery, radiation, targeted therapy, and hormonal therapy. We also confirm better results in patients with earlier stages and support awareness and early detection in LMICs as well. As for access to newer therapeutic agents, medications are usually made available in Lebanon after they are approved by the (US) Food and Drug Administration and/or the European Medicinal Agency. Governmental and private sector employees are generally covered under various public health insurance plans including National Social Security Fund and private insurances.^15,16^ Medications that were available at the AUBMC were also available throughout the country for all patients.

Our results indicate that the prognosis and outcome of young women with breast cancer improve with modern multidisciplinary management that includes neoadjuvant/adjuvant therapy, surgery, radiation, targeted therapy, and hormonal therapy according to their tumor characteristics and stage. Newer modalities of treatment have more recently also shown improvement in the outcome and survival of patients with breast cancer. Clinical trials with the new anti-HER2 agents in the neoadjuvant and adjuvant settings and metastatic settings in HER2-positive disease,^17-20^ cyclin-dependent kinase 4/6 inhibitors in the metastatic and adjuvant setting in hormone receptor–positive disease,^21^ immunotherapy in the metastatic and neoadjuvant setting in TNBC,^22,23^ and poly (ADP-ribose) polymerase inhibitors in patients with germline *BRCA* mutations^24^ have shown improvement in survival of patients with breast cancer.

In conclusion, we report high survival rates in young patients diagnosed below age 40 years with breast cancer treated with modern multidisciplinary management. We report survival rates of 89% at 5 years and 76% at 10 years. Best results are seen in patients with early breast cancer; patients with stages I and II had an OS of 96.0% at 5 years and 87.1% at 10 years, whereas patients with stage III had an OS of 77.0% at 5 years and 66.0% at 10 years.

Modern multidisciplinary management, guided by biology and modern therapies, can help us remove the stigma of poor prognosis in young women with breast cancer. Our data, coming from a LMIC, show that improvement is achievable everywhere when resources are available and treatment is accessible.

## References

[1] Sung H Ferlay J Siegel RL et al Global cancer statistics 2020: GLOBOCAN estimates of incidence and mortality worldwide for 36 cancers in 185 countries CA Cancer J Clin 71 209 249 2021 33538338 10.3322/caac.21660

[2] Partridge AH Hughes ME Warner ET et al Subtype-dependent relationship between young age at diagnosis and breast cancer survival J Clin Oncol 34 3308 3314 2016 27480155 10.1200/JCO.2015.65.8013

[3] Assi HA Khoury KE Dbouk H et al Epidemiology and prognosis of breast cancer in young women J Thorac Dis 2013 5 S2 S8 suppl 1 23819024 10.3978/j.issn.2072-1439.2013.05.24 PMC3695538

[4] Clarke CA Keegan TH Yang J et al Age-specific incidence of breast cancer subtypes: Understanding the black-white crossover J Natl Cancer Inst 104 1094 1101 2012 22773826 10.1093/jnci/djs264 PMC3640371

[5] Gabriel CA Domchek SM Breast cancer in young women Breast Cancer Res 12 212 2010 21067532 10.1186/bcr2647 PMC3096966

[6] El Saghir NS Khalil MK Eid T et al Trends in epidemiology and management of breast cancer in developing Arab countries: A literature and registry analysis Int J Surg 5 225 233 2007 17660128 10.1016/j.ijsu.2006.06.015

[7] El Saghir NS Shamseddine AI Geara F et al Age distribution of breast cancer in Lebanon: Increased percentages and age adjusted incidence rates of younger-aged groups at presentation J Med Liban 50 3 9 2002 12841305

[8] El Saghir NS Seoud M Khalil MK et al Effects of young age at presentation on survival in breast cancer BMC Cancer 6 194 2006 16857060 10.1186/1471-2407-6-194 PMC1555600

[9] Azim HA Jr Michiels S Bedard PL et al Elucidating prognosis and biology of breast cancer arising in young women using gene expression profiling Clin Cancer Res 18 1341 1351 2012 22261811 10.1158/1078-0432.CCR-11-2599

[10] Keegan THM Press DJ Tao L et al Impact of breast cancer subtypes on 3-year survival among adolescent and young adult women Breast Cancer Res 15 R95 2013 24131591 10.1186/bcr3556 PMC3978627

[11] Anderson BO Cazap E El Saghir NS et al Optimisation of breast cancer management in low-resource and middle-resource countries: Executive summary of the Breast Health Global Initiative consensus, 2010 Lancet Oncol 12 387 398 2011 21463833 10.1016/S1470-2045(11)70031-6

[12] Anderson BO Ilbawi AM El Saghir NS Breast cancer in low- and middle-income countries (LMICs): A shifting tide in global health Breast J 21 111 118 2015 25444441 10.1111/tbj.12357

[13] El Saghir NS Farhat RA Charara RN et al Enhancing cancer care in areas of limited resources: Our next steps Future Oncol 10 1953 1965 2014 25386812 10.2217/fon.14.124

[14] Frerichs RR: Rapid surveys. 2008. https://www.ph.ucla.edu/epi/rapidsurveys/RScourse/RSbook_ch3.pdf

[15] Wolff AC Hammond MEH Allison KH et al Human epidermal growth factor receptor 2 testing in breast cancer: American Society of Clinical Oncology/College of American Pathologists clinical practice guideline focused update J Clin Oncol 36 2105 2122 2018 29846122 10.1200/JCO.2018.77.8738

[16] Amin MB Greene FL Edge SB et al The Eighth Edition AJCC Cancer Staging Manual: Continuing to build a bridge from a population-based to a more “personalized” approach to cancer staging CA Cancer J Clin 67 93 99 2017 28094848 10.3322/caac.21388

[17] Ministry of Public Health of Lebanon, TOKTEN, Counsel on Development and Reconstruction, United Nations Development Program (UNDP) National cancer treatment guidelines 2012 https://www.moph.gov.lb/en/Pages/4/4768/drugs-national- guidelines

[18] Elias F Khuri FR Adib SM et al Financial burden of cancer drug treatment in Lebanon Asian Pac J Cancer Prev 17 3173 3177 2016 27509947

[19] Piccart M Procter M Fumagalli D et al Adjuvant pertuzumab and trastuzumab in early HER2-positive breast cancer in the APHINITY trial: 6 years' follow-up J Clin Oncol 39 1448 1457 2021 33539215 10.1200/JCO.20.01204

[20] Swain SM Miles D Kim SB et al Pertuzumab, trastuzumab, and docetaxel for HER2-positive metastatic breast cancer (CLEOPATRA): End-of-study results from a double-blind, randomised, placebo-controlled, phase 3 study Lancet Oncol 21 519 530 2020 32171426 10.1016/S1470-2045(19)30863-0

[21] Conte P Schneeweiss A Loibl S et al Patient-reported outcomes from KATHERINE: A phase 3 study of adjuvant trastuzumab emtansine versus trastuzumab in patients with residual invasive disease after neoadjuvant therapy for human epidermal growth factor receptor 2-positive breast cancer Cancer 126 3132 3139 2020 32286687 10.1002/cncr.32873 PMC7317721

[22] Modi S Saura C Yamashita T et al Trastuzumab deruxtecan in previously treated HER2-positive breast cancer N Engl J Med 382 610 621 2020 31825192 10.1056/NEJMoa1914510 PMC7458671

[23] Tripathy D Im SA Colleoni M et al Ribociclib plus endocrine therapy for premenopausal women with hormone-receptor- positive, advanced breast cancer (MONALEESA-7): A randomised phase 3 trial Lancet Oncol 19 904 915 2018 29804902 10.1016/S1470-2045(18)30292-4

[24] Rizzo A Cusmai A Acquafredda S et al KEYNOTE-522, IMpassion031 and GeparNUEVO: Changing the paradigm of neoadjuvant immune checkpoint inhibitors in early triple-negative breast cancer Future Oncol 18 2301 2309 2022 35378995 10.2217/fon-2021-1647

